# Wireless wearable devices for continuous monitoring of body sounds and motions

**DOI:** 10.1002/ctm2.1593

**Published:** 2024-02-16

**Authors:** Sun Hong Kim, Emily Jeanne, Wissam Shalish, Jae‐Young Yoo, John A. Rogers

**Affiliations:** ^1^ Querrey Simpson Institute for Bioelectronics Northwestern University Evanston Illinois USA; ^2^ Neonatal Division Department of Pediatrics McGill University Health Center Montreal Quebec Canada; ^3^ Department of Semiconductor Convergence Engineering Sungkyunkwan University Suwon Republic of Korea; ^4^ Department of Biomedical Engineering Northwestern University Evanston Illinois USA; ^5^ Department of Materials Science and Engineering Northwestern University Evanston Illinois USA; ^6^ Department of Neurological Surgery Northwestern University Chicago Illinois USA

## 1 INTRODUCTION

Diverse body processes such as those related to respiration and digestion generate characteristic sounds and mechanical vibrations.^1^, ^2^, ^3^ Quantitative measurements of these phenomena hold invaluable clinical information regarding activity of the cardiopulmonary system, flow of air into/out of the lungs and blood through arteries/veins, ingestion and swallowing of liquid and solids, and motility of matter through the stomach and intestines.^4^, ^5^, ^6^ Historically, the stethoscope has played a crucial role in the capture of these body sounds for diagnosis of cardiovascular and respiratory diseases. In traditional passive analogue and active digital systems, the rigid designs and relatively large sizes of these devices represent drawbacks that prevent their use on challenging regions of the body and on small, vulnerable patients such as premature infants. Susceptibility to ambient sounds and motion artefacts are additional considerations that confine applications to controlled environments over short measurement periods, thereby creating challenges for home use and for continuous monitoring. Additional limitations are in the inability to perform simultaneous recordings from multiple body locations, thus necessitating a point‐by‐point serial evaluation process that fails to capture precise time synchrony in sound or motion characteristics.

**FIGURE 1:**
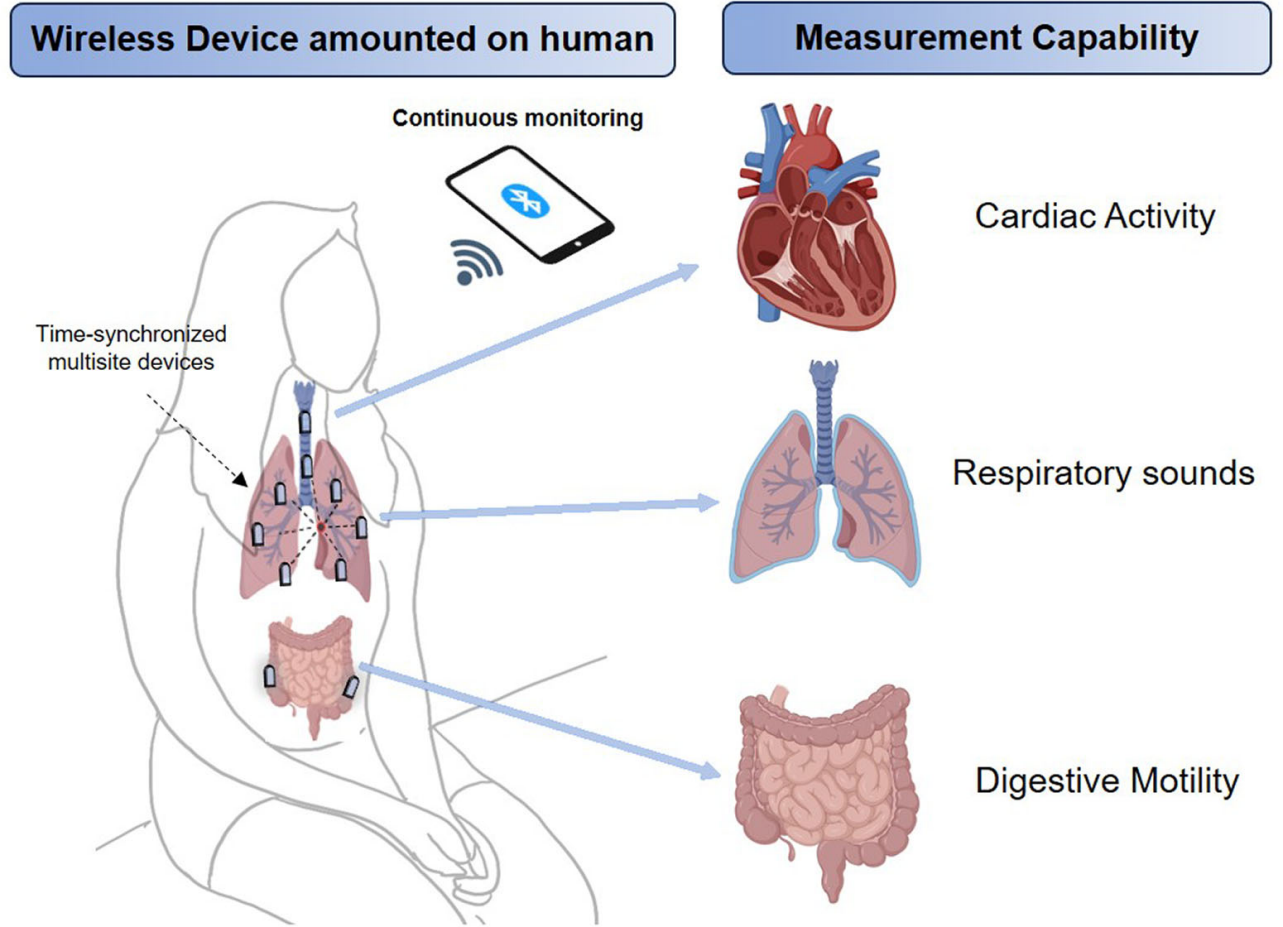
Wireless, miniaturised sensors of body sounds and motion for continuous physiological monitoring and diagnostics. Schematic illustration showing time‐synchronised multi‐sited devices are mounted on human skin for tracking cardiorespiratory activity, multi‐location respiratory sounds and gastrointestinal motility.

Recent reports describe advances in miniaturised, soft electronic devices designed for sensing subtle body vibrations and sounds, with additional capabilities in tracking bulk motions of the body and noises^5^, ^7^, ^8^, ^9^, ^10^ in the ambient environment through the use of multi‐sensor designs and sound separation data analytics, respectively (Figure 1).^11^ As implemented with wireless strategies in time synchronisation and data communication, these small, skin‐interfaced sensors enable continuous monitoring of not only sounds and motions related to respiratory and digestive activity, but also core vital signs, such as heart and respiratory rates, across multiple key body locations in both infants and adults. The resulting information can facilitate assessments of patient status, beyond the possibilities of conventional measurements, in both clinical and home settings. Specific examples include spatiotemporal mapping of the dynamics of gastrointestinal processes and the patterns of air flow into and out of the lungs.

## 2 CONTINUOUS MONITORING OF RESPIRATORY ACTIVITY IN NEONATES

Premature infants hospitalised in the Neonatal Intensive Care Unit are at risk of cardiorespiratory instabilities due to immature respiratory control and airway obstruction.^12^, ^13^ These conditions typically cause cessation of breathing (apneas) accompanied by abnormal fluctuations in heart rate and oxygen saturation. Unfortunately, existing devices for detecting airway obstruction have drawbacks, including bulky, wired designs that are susceptible to noise and movement artefacts during basic clinical operations, and are incompatible with nasal interfaces. Our recent work addressed these limitations through the development of a wireless broadband acousto‐mechanical sensing (BAMS) system that enables the detection of body sounds and motion for continuous physiological monitoring and diagnostics for this clinical use case.

In our study, we showed that the BAMS system can capture respiratory sounds during periods of normal, restricted and absent airflow in hospitalised premature neonates. The data can also quantify respiratory sound intensities and compute respiratory rates with high fidelity compared to FDA‐approved clinical monitoring devices. In addition to facilitating the continuous monitoring of breathing, the same system can capture a comprehensive range of other parameters, including heart rate, ambient noise and body rotation angles. The results enable simultaneous tracking of cardiac activity, external auditory stimuli, and patient positioning and movements. This versatility has significant potential for identifying risks in premature infants with cardiorespiratory instability, thereby offering valuable clinical insights and alleviating stress for caregivers.

## 3 SPATIOTEMPORAL MONITORING OF DIGESTION IN NEONATES

The sounds resulting from the movement of food, gas and fluids during intestinal peristalsis hold key information for understanding and diagnosing gastrointestinal health.^14^, ^15^ In fact, they are routinely auscultated in the acute care setting as part of clinical standards, to rule out various intestinal diseases and motility disorders across different age groups. Auscultation of bowel sounds using a traditional stethoscope can, however, only realistically be conducted for a short period at a single location with only a subjective interpretation of the auditory input. By contrast, continuous, wireless, multi‐location monitoring of bowel sounds is possible with the BAMS system placed over the abdomen. Data analytics and real‐time continuous data streaming with a time‐synchronised set of devices provide access to normalised sound intensity from each site, yielding spatiotemporal information related to intestinal motility. These features are particularly attractive in the clinical setting, as they have the potential to be utilised as an early warning system for detecting impending gastrointestinal complications. One such example in neonates is necrotising enterocolitis, a devastating disease characterised by intestinal inflammation and ischaemia, which typically manifests acutely with abdominal distension, reduced peristalsis and feeding intolerance. Application of the BAMS system in such a population may be beneficial, especially considering that prompt diagnosis and treatment are paramount for minimising the risk of bowel necrosis.

## 4 SPATIOTEMPORAL MONITORING OF AIR FLOW IN PATIENTS WITH PULMONARY DISEASE

The BAMS technology facilitates simultaneous capture of lung sounds and body motions across various anatomical locations, as the basis for an innovative perspective in respiratory diagnostics. This approach provides a comprehensive analysis of a single breath at multiple lung regions, with substantial promise for enhancing the diagnosis and monitoring of diverse lung pathologies. The utilisation of BAMS devices on the anterior and posterior chest allows for the establishment of a clear link between spatial information and the sound intensity and frequency components of the acoustic signals. Such integration of imaging and sound data holds promise as a valuable diagnostic tool that may improve the precision in identification of specific pulmonary conditions. Particularly noteworthy is a comparative analysis involving patients with chronic lung diseases who underwent surgical lung resections. The identified variations in sound intensity ratios and dominant expiratory frequencies present promising markers for evaluating the impact of surgical interventions on lung function. This insightful information contributes to a nuanced comprehension of post‐operative outcomes, offering valuable guidance for personalised treatment plans.

## 5 FUTURE PROSPECTS FOR CONTINUOUS BODY SOUND MONITORING

Results obtained with the BAMS system showcase diverse capabilities in continuous monitoring of acoustic signals and motion events. The use of real‐time, automated algorithms for data analytics have potential to improve the efficiency, precision and objectivity of clinical monitoring across various health care sectors, populations and conditions. As one of many examples, the continuous assessment of intestinal motility following gastrointestinal surgery may sidestep the constraints of nurse availability and subjective interpretation of auscultated bowel sounds, thus enabling the timely development of postoperative feeding plans. Furthermore, the time‐synchronised, multi‐location features of the BAMS system are important assets with significant implications in critically ill patients. In the latter, capturing a real‐time reduction in airflow confined to one side of the chest could be integral for early diagnosis and management of localised conditions such as lung collapse, consolidation and pneumothorax, thereby minimising lung injury and improving outcomes. Finally, the wireless, wearable nature of the BAMS devices minimises patient discomfort and facilitates remote diagnostic procedures. Namely, stable patients referred to the sleep lab for suspected apnoea could be eligible for screening at home, unincumbered by intrusive wires, which further helps to preserve their natural sleep patterns. In addition, some hospitalised patients may be prime candidates for earlier discharge and at‐home monitoring during convalescence, which could reduce length of stay and curtail hospitalisation costs. As technologies such as the BAMS system continue to emerge and improve, high‐quality studies will be needed to evaluate their safety and clinical usefulness in the most vulnerable patients.

## CONFLICT OF INTEREST STATEMENT

The authors declare they have no conflicts of interest.
